# Perceptions of Obesity and Treatment Decision‐Making Among Hispanic‐Latino Adults: Barriers to Metabolic and Bariatric Surgery

**DOI:** 10.1002/osp4.70156

**Published:** 2026-06-18

**Authors:** Viviane Fornasaro‐Donahue, Ceren Gunsoy, Kathleen J. Melanson

**Affiliations:** ^1^ Department of Psychology University of Rhode Island Providence Rhode Island USA; ^2^ Department of Psychology, Behavioral Sciences University of Rhode Island Kingston Rhode Island USA; ^3^ Department of Nutrition, Human Energy Balance Laboratory University of Rhode Island Kingston Rhode Island USA

**Keywords:** hispanic‐latino, metabolic and bariatric surgery, obesity

## Abstract

**Objective:**

Metabolic and Bariatric Surgery (MBS) is underutilized among Hispanic‐Latino adults despite higher obesity rates and related comorbidities. Although barriers such as cost, language, and limited provider referrals are documented, sociocultural and psychological perceptions about obesity and obesity care remain less understood. This study aimed to explore the beliefs, experiences, and information sources influencing perceptions of obesity, obesity treatment, and patient‐provider communication, and how these factors relate to MBS decision‐making in this population.

**Methods:**

Semi‐structured, in‐depth interviews were conducted with 14 Hispanic‐Latino adults in Rhode Island who met the eligibility criteria for MBS. Interviews were transcribed, reviewed, and thematically analyzed using an inductive approach to identify patterns in obesity perceptions, treatment beliefs, and healthcare communication.

**Results:**

Participants expressed fear, skepticism, and misinformation about MBS, shaped by stigma, cultural values, and personal beliefs. Many viewed MBS as a last resort and cited concerns about complications or long‐term effects. Participants often relied on informal sources—such as family and friends—for obesity treatment information rather than healthcare providers. Communication with providers about obesity treatment options was often limited or non‐specific.

**Conclusion:**

Findings highlight the need for culturally tailored bilingual education and improved patient‐provider communication to increase MBS awareness and trust. Addressing these gaps may help reduce disparities in obesity care access among Hispanic‐Latino adults.

## 1 Introduction

Obesity affects racial groups differently, with higher prevalence among minorized groups, particularly among non‐Hispanic Black adults (49.9%) and Hispanic‐Latino individuals (45.6%) [1]. Although Metabolic and Bariatric Surgery (MBS) is an effective long‐term treatment for individuals with obesity, in general only 1% of all eligible people utilize surgery [2], and it is especially underutilized in the Hispanic‐Latino population [3]. National hospital data indicated that 70% of individuals who undergo MBS are non‐Hispanic White, 15% are non‐Hispanic Black, and only 10% are Hispanic individuals [4].

In addition to the higher prevalence of obesity, the Hispanic‐Latino population presents with a higher prevalence of related comorbidities, such as cardiovascular disease, dyslipidemia, non‐alcoholic liver disease and type 2 diabetes, compared with the White population [5]. MBS is associated with improvements in these obesity‐related conditions as well as hypertension, sleep apnea, gastroesophageal reflux, and others [6]. The higher prevalence of obesity, the presence of related comorbidities, and the lower accessibility of weight management interventions for the Hispanic‐Latino population [7, 8, 9] underscore the importance of focusing on access and utilization of obesity treatment options among this population [9, 10, 11].

A recent review of obesity among Hispanic‐Latino individuals in the U.S. identified multiple contributing factors to the increased prevalence of obesity in this population [5]. These include genetic‐susceptibility, dietary patterns, socioeconomic status, culturally influenced body‐ideas, environmental factors, and health care provider‐related factors, such as language barrier and lack of insurance coverage [5]. Furthermore, systemic barriers—including cost, limited insurance coverage, and referral biases—have been recognized in the literature as contributors to the underutilization of MBS among Hispanic‐Latino individuals [5, 12, 13]. However, there remains a lack of in‐depth examination of the socio‐cultural factors influencing MBS utilization, highlighting the need for further exploration [14, 15].

This study aimed to explore the socio‐cultural factors influencing the underutilization of MBS among Hispanic‐Latino adults with obesity living in the U.S. Specifically, we sought to deepen our understanding of individuals' perspectives on obesity and obesity care, including MBS, obesity management medication (OMM), and patient‐provider communication, which can influence decision making regarding MBS. We expected that understanding this community's perceptions and needs related to obesity‐care can narrow the MBS utilization gap. This information could support providers in personalizing obesity care by incorporating the voices of those directly affected and fostering culturally sensitive communication with this population.

## 2 Methods

The study utilized qualitative design through semi‐structured in‐depth interviews to explore the perceptions of obesity, obesity care, and MBS among Hispanic‐Latino individuals who fall into the eligibility category for MBS. It was approved by the Institutional Review Board of the University of Rhode Island. The study was also funded by the University of Rhode Island.

### 2.1 Participants

Fourteen Hispanic‐Latino individuals from a community center located in Providence, Rhode Island, who met the MBS eligibility criteria were recruited. The recruitment involved the distribution of flyers and networking with community leaders. Eligibility criteria included being from a Hispanic‐Latino background, having obesity, and being 18 years old or older. A demographic questionnaire was used to collect participants' anthropometric data (height, weight, and BMI) and obesity‐related conditions, including diabetes, prediabetes, sleep apnea, hypertension, and gastroesophageal reflux disease. All participants classified as having Class I (BMI 30 to ≤ 35 kg/m^2^) obesity were screened to ensure they met eligibility based on the presence of co‐morbidities consistent with the American Society Metabolic and Bariatric Surgery recommendations—MBS is recommended for individuals with a BMI ≥ 35 kg/m^2^, regardless of presence, absence, or severity of co‐morbidities, BMI ≥ 30 kg/m^2^ with Type II Diabetes, and should be considered in individuals with BMI of 30–34.9 kg/m^2^ with metabolic disease who do not achieve substantial or durable weight loss or improvement in obesity‐related conditions through nonsurgical methods [16]. Participants were asked to select the group that best described their Hispanic‐Latino cultural background and to indicate their country of origin. Participants were assigned unique numerical identification codes, and no personal identifiers were linked to their data to ensure confidentiality.

### 2.2 Materials and Procedure

Following the verification of eligibility, participants were scheduled for an approximately 30‐min in‐person interview with the researcher, with the session being recorded. To express appreciation for their time and contribution, participants received a $25 gift card. Interviews were conducted in English or Spanish by the researcher, with the assistance of a Spanish speaking staff member when necessary. Prior to the interview, the consent form was reviewed, and verbal consent was documented.

The researcher initiated the recording and adhered to the semi‐structured script when conducting interviews with participants. Participants were also asked a range of questions tailored to their social and cultural characteristics (e.g., country of origin, length of residence in the U.S., socioeconomic status, and education level), anthropometrics (including self‐reported height and weight), presence of obesity‐related conditions and acculturation (outlined in Table 1). The acculturation measures included the Psychological Acculturation Scale (PAS), which examines how individuals navigate between two cultural identities [17], and the Short Acculturation Scale for Hispanics (SASH), which explores language usage [18]. These measures aim to capture the sociocultural orientations that individuals develop while living within a new cultural system.

**TABLE 1:** Participant characteristics of a community sample of hispanic‐latino adults.

Characteristics (*n* = 14)	Frequency (*n*)	Percent (%)
Body Mass index (BMI) class		
— Obesity class 1 (30 to < 35)	10	71.4
— Obesity class 2 (35 to < 40)	3	21.4
— Obesity class 3 (40 or greater)	1	7.1
Presence of obesity‐related condition(s)—prediabetes, diabetes type II, hypertension, gastroesophageal reflux disease and or sleep apnea		
— Yes (at least one obesity‐related condition or more)	12	85.7
Country of origin		
Colombia	1	7.1
Guatemala	1	7.1
Cuba	1	7.1
Dominica Republic	4	28.6
Puerto Rico	7	50.0
Language(s) do you read/speak/think		
Only English	1	7.1
English Better than Spanish	3	21.4
Both equally	5	35.7
Spanish Better than English	3	21.4
Only Spanish	2	14.3
With which group(s) of people do you feel you share most of your beliefs, traditions, feel most comfortable/proud, and understands?		
Only Hispanic/Latino	3	21.4
Mostly Hispanic/Latino	7	49.8
Equally	3	21.4
Mostly White American	1	7.1

Interviews were transcribed using OtterAI, a transcription software capable of processing audio recordings in both Spanish and English. The lead researcher, who is bilingual, listened to all recordings, reviewed every transcript to ensure completeness, and removed all personal identifiers. A bilingual research assistant reviewed all transcripts and translated the Spanish transcripts into English. The translated transcripts were returned to the lead researcher to verify the fidelity and integrity of the data through back‐translation. This process spanned approximately 2 months (April–June 2025) and involved multiple reviews of each transcript by both researchers to ensure that the content was accurately transcribed and contextually appropriate.

The analysis of the interview data was conducted manually by the research team, following the thematic analysis framework outlined by Braun and Clarke (2006). Both the lead researcher and the bilingual research assistant independently read all transcripts multiple times—first for familiarization and then question by question—to identify and code recurring concepts. Their results were then compared and reconciled to produce a unified set of themes and subthemes. Each theme was carefully reviewed to ensure internal coherence and alignment with the overall data set. The themes were then defined and named with continuous refinement. The analysis was conducted using an inductive approach, meaning that themes were derived directly from the data without attempting to fit responses into pre‐existing codes or theoretical frameworks. Participant quotes were extracted from the interviews to illustrate and support the identified themes, providing direct insight into the participants' experiences and perspectives. Weekly meetings with the lead researcher, research assistant, and co‐authors were held throughout the analytic process to discuss emerging themes, quotes, and interpretation, and to finalize the thematic framework.

## 3 Results

### 3.1 Participants' Characteristics

Additional characteristics of 14 Hispanic‐Latino adults are summarized in Table 1. Participants were predominantly women (85.7%) with a mean age of 44 years and an average BMI in the Class I obesity range (*M* = 34.11 ± 2.49 kg/m^2^). Most (85%) had obesity‐related health conditions and had lived in the United States for an average of 31.43 years (range: 10–52). The majority had at least a high school education, with 50% holding a high school diploma, 35.7% having some college education, and a small proportion (14.2%) completing trade school or less than eighth grade. Most participants identified as Christian (85.7%), and household income was generally low, with 71.4% reporting annual incomes below $30,000. Half of the participants were single (50%), and half were married or partnered (50%). Most were unemployed (71.4%) and reported having public insurance coverage (71%).

### 3.2 Semi‐Structured Interviews

Table 2 presents themes and representative quotes. The key findings are presented within three main topics: (1) Participants' perception of obesity, (2) MBS perception, and (3) Patient‐provider communication. Within these three main topics, themes were generated and labeled as follows: 1.a to 1.e, 2.a to 2.e, and 3.a to 3.d. As shown in the next sections.

**TABLE 2:** Key themes with corresponding quotations from semi‐structured interviews with Hispanic‐Latino adults with obesity.

1. Overall theme: obesity perception
1. a) Participants' perceptions of individuals living with obesity and their self‐perceptions as individuals living with obesity include psychological, behavioral, physical, social, and health‐related domains
“Most of them suffer. They feel shame… they got a lot of depression.” “I feel they are neglecting themselves. “The first thing you see is their body, you see they are overweight, you can see how the person walks, like they are getting tired.” “Frustrated. Depressed.” “I feel sleepy… I have a weight that makes me not want to do anything.” “Health problems”
1. b) Participants' perceived causes of obesity in others and of own obesity include psychological, genetic, behavioral, eating‐related, physical, and health‐related factors.
“Maybe they are going through anxiety or depression. “The environment, stress…” “Eating too much, eating badly…not doing any type of exercise.” “Mostly depression, lack of support at home.” “the problem is eating at night. If you eat a plate of rice, plantains and meat at night.” “It's the street food, like burger king and McDonalds, all that.” “I think it's inherited.” “Not exercising enough”
1. c) Participants' reported mixed views of obesity as chronic disease
“We sit, keep eating, we don't take care of ourselves, we don't eat healthy things. The problem with us hispanics, is that we love rice.” “I've tried but I can't, I keep gaining weight every day—so I think it could be a disease.”
1. d) Participants' perception of individuals without obesity include psychological, physical, health‐related, behavioral, and social‐environmental domains
“Maybe happier because everything fits well, they feel more confident.” “Someone calm” “Maybe more active, physically and socially, less stressed.” “have a better living condition, environment around them, maybe they have people who are supportive.”
1. e) Participants' thoughts about obesity treatment apart from MBS include nutrition programs, obesity management medications, and exercise
“I feel like these nutrition programs; they are mostly for profit.” I'm not convinced because all medications have consequences. It may fix one thing, but it damages something else in your system.” “I stay on my end of fitness journey, and I don't believe in such thing as diet.”
2. Overall theme: perception of metabolic and bariatric surgery
2. a) Participants' mixed perceptions of MBS reflect negative views, with some perceived benefits.
“If you want to change your physical appearance, you should set your mind to do it yourself. I don't agree with having surgery.” “It seems like a risk to me. I wouldn't want it for myself, no. I think people shouldn't have to go that far.” “I think pretty much a surgery that's going to help somebody get their life back.”
2. b) Participants understanding of or knowledge about MBS include pre‐surgical requirements, surgical procedure, cost, effectiveness
“I know how you need to lose (or gain) a certain amount of weight before you do the surgery. You need to get approval from all your specialists.” “After care is very important, going to the gym, and your diet…” The support at home is a must” “It's a little operation. It's only five incisions, but two, 3 years, that's still open inside”. “They cut the stomach, right? Or they put a band there or something like that.” “I have a friend who had it done…she kept eating, and now she is even more overweight”
2. c) Sources of awareness of MBS are primarily friends, family, and personal networks
“I heard about it when I was younger. My mom started the process when I was 17.” “I know a lady…”
2. d) Participants' attitudes toward MBS recommendation for self and others range from acceptance to opposition, including conditional openness.
“If I were to get it [surgery], I feel like I will get a lot of criticism, so I would have to prepare myself before getting the surgery to handle all of the unnecessary or unsolicited opinions.” “Thinking about surgery for myself, it doesn't really make sense, no.” “Thinking about surgery for myself, it doesn't really make sense, no.” “I would probably encourage them. I know that in every surgery there's risk, but if it's suitable for them, and make them feel better and happier than, yeah, sure”. “That they should think it over and first try exercise and healthy eating.”
2. e) Anticipated life changes resulting from weight management include psychological, social, and health‐related domains
“It could do a 360 change in me, physically and entirely because me being bigger now, I'm less active.” “Medically, maybe I can better my A1C. I can maybe get rid of my asthma and my COPD.” “I can walk more. Qualify for knee surgery.”
3. Overall theme: patient‐provider communication
3. a) Communication experiences regarding weight concerns encompass trust, engagement, communication style, language, and care coordination
“He [doctor] says, “You're overweight, you need to eat other things,” but he never offered injections, pills, or bariatric surgery. “They changed my primary care doctor three times in the last year and I've been having problems, because they don't know me—I don't have the same confidence.” “The patient has to decide to talk with the doctor. If they don't, the doctor can do nothing.” “some doctors they tiptoe around it, just so they can avoid hurting your feelings” “They just say ‘yes, exercise,’ and that's it. They order tests, but they don't seem truly concerned.” He [doctor] doesn't speak Spanish, and I don't speak English, but we understand each very well.” “they haven't told me they are going to send me to a nutrition specialist.”
3. b) Considerations and motivations for pursuing MBS include post‐partum weight retention, social support, and peer success stories.
“If after having my kids, if I don't take care of myself with the pregnancy.” “For somebody to go with me, to go to the surgery. I cannot go by myself.” “Motivating me would be seeing others results or haring about other results.”
3. c) Perceived barriers to pursuing MBS include surgical risks and personal beliefs
“Something that's going to stop me would be the risks involved” “I wouldn't like [surgery]. Sometimes I think about surgery for my breasts, but I stay with them like that because that's how god gave them to me.”
3. d) Information needs related to obesity care and MBS include communication, lifestyle modifications, sociocultural considerations, eligibility criteria, and resources.
“Maybe having more accessible bilingual providers or interpreting services.” You got to adapt to a lot of things, and specially all Spanish people, like christmas time, we got to eat.” “the drinking, the people that drink, that smoke, you got to change your whole life.” “Brochures, TV commercials and word of mouth.” “What level of obesity you have to have to qualify for bariatric surgery.”

Abbreviations: MBS = metabolic and bariatric surgery; OMM = obesity management medication.

### 3.3 Perception of Obesity

When asked to describe individuals with obesity, participants shared perceptions shaped by emotional, behavioral, physical, social, and health‐related observations. Emotionally, individuals with obesity were frequently associated with depression, shame, and embarrassment. Several participants expressed sympathy, noting that people with obesity “suffer” emotionally or feel judged by others. Behaviorally, many described individuals with obesity as overeating, lacking control over their diet, or neglecting healthy habits. Physically, participants referenced visible traits such as being “chubby,” “large,” or “sluggish,” and mentioned difficulties with mobility and daily functioning. Socially, some expressed embarrassment or misfitting. Health‐related perceptions included assumptions about fatigue, risk of collapse, and general physical strain due to excess weight, with participants commonly identifying individuals with obesity as “unhealthy,” “without health,” or facing ongoing “health problems.” Overall, participants' responses reflected a combination of stigmatizing beliefs, personal empathy, and internalized societal views about people living with obesity (Table 2: 1.a).

Participants identified several perceived causes of obesity in others, reflecting a range of psychological, behavioral, physical, genetic, and health‐related explanations. Psychologically, many attribute obesity to depression, anxiety, stress, and emotional instability, often linking these conditions to behaviors such as overeating, constant snacking, or staying at home and being inactive. Some participants described stressful environments as contributing to poor mental health and, in turn, weight gain. Genetically, only one participant mentioned that obesity could be inherited. Dietary choices and eating behaviors were frequently cited, including excessive consumption of “junk food,” unhealthy eating habits, eating late at night, and a lack of balanced meals. Health‐related causes included the use of certain medications and experiences with heart conditions. Some participants emphasized physical inactivity as a key contributor, noting behaviors such as not exercising, sitting for long periods, or recovering from an injury. A few also suggested that obesity may result from personal neglect or lack of care. Overall, the responses reflect a mix of psychological, behavioral and personal beliefs with varying degrees of empathy and judgment (Table 2: 1.b).

Participants expressed mixed views on whether obesity should be considered a chronic disease. Some participants disagreed with the classification, emphasizing that obesity could be controlled through personal responsibility, lifestyle changes, and following medical guidance. They suggested that with proper self‐care and motivation, individuals could prevent or reverse obesity. Others expressed agreement with the idea that obesity is a chronic condition, noting the difficulty many people face in losing weight despite repeated efforts. Participants described obesity as persistent. Some also acknowledged that obesity can lead to serious health complications and, depending on its severity, may be life‐threatening. These responses illustrate a division in perspectives: while some viewed obesity as preventable and manageable by one's responsibility, others recognized it as a complex, ongoing condition that can be difficult to treat (Table 2: 1.c).

Participants described individuals without obesity in largely positive terms, highlighting emotional, physical, health‐related, behavioral, and social attributes. Emotionally, they were perceived as calm, confident, and possibly happier, particularly due to factors like clothing fitting well and reduced stress. Physically, participants described them as having bodies in proportion to their height and weight, with greater flexibility and energy. Health‐related perceptions included a lower risk of illness, increased longevity, and overall better physical health. In terms of dietary behaviors, individuals without obesity were viewed as having self‐control, following a balanced diet, eating at appropriate times, and avoiding foods that could lead to conditions like diabetes. Socially, some participants believed that individuals without obesity were more active and had better support systems or living conditions. While most responses reflected a clear contrast between individuals with and without obesity, one participant noted that “everyone got something going on,” suggesting that weight alone does not define a person's overall well‐being (Table 2: 1.d).

Participants reflected on their personal experiences living with obesity, offering insights into its emotional, physical, and health‐related impacts. Emotionally, many expressed feelings of depression, frustration, low self‐esteem, and discomfort in their own bodies. Some described a sense of emotional growth after weight loss, reporting improved self‐esteem, relief, and increased confidence. Others shared ongoing struggles with body image, noting discomfort when looking in the mirror or dissatisfaction with their appearance. Physically, participants reported feeling tired, bloated, and less active, with some describing a lack of motivation or difficulty engaging in daily activities due to their weight. Health‐related self‐perceptions included awareness of chronic conditions such as asthma and acknowledgment of the potential consequences of excess weight. These responses illustrate how living with obesity affected participants not only physically but also emotionally and psychologically, shaping their self‐concept and daily experience (Table 2: 1.a).

Participants identified a range of perceived causes for their own obesity, including psychological, behavioral, and genetic factors. Psychologically, many attributed their weight gain to depression, anxiety, and mental health challenges. These emotional struggles were at times linked to patterns of overeating or loss of control around food. Behaviorally, participants pointed to specific eating habits such as late‐night meals, frequent consumption of high‐calorie foods, fast food, or “street food”, and large portions. In addition to dietary behaviors, some participants acknowledged a lack of physical activity or exercise as a contributing factor. Genetically, one participant believed that obesity might be inherited, noting that their body size had always been larger, despite not eating excessively. These self‐assessments reflect a nuanced understanding of obesity as a result of both internal and external influences, with emotional well‐being and lifestyle seen as contributing elements (Table 2: 1.b).

Participants shared a variety of perspectives on obesity treatments outside MBS, primarily focusing on diet, physical activity, and less frequent OMM. Most participants discussed dietary changes as a central method of weight management. Some emphasized moderation, portion control, and healthy choices, such as eating fruits, drinking green juice, or “juice shots,” and cutting processed foods. While several participants saw diet as a strategy, a few were skeptical of commercial diet programs, viewing them as profit‐driven or unnecessary with self‐discipline. Physical activity was also mentioned as a key component, described as a personal commitment and lifestyle.

Participants' opinions on OMM were mixed. Two participants found OMM effective in reducing appetite and expressing satisfaction. Others voiced concerns about side effects, long‐term consequences, and drug interactions. Some had never heard of OMM or were uncertain about its purpose or safety. Accessibility also emerged as a concern, with barriers such as insurance coverage and out‐of‐pocket costs (Table 2: 1.e).

### 3.4 Perception of Metabolic and Bariatric Surgery

Participants primarily expressed caution, skepticism, or concerned views toward MBS, with a few expressing supports. Many were opposed to MBS, and some viewed it as a last resort or unnecessary, citing fear of complications, side effects, or long‐term health issues. Some referenced negative experiences of others, such as difficulty eating, pain, or additional surgeries, or believed that weight loss should come from self‐discipline. The perception that MBS requires strong mental preparation, distrust in medical procedures, and not considering their weight as “extreme” were also barriers. A few viewed MBS as risky, unnatural, or dangerous citing medical errors or deaths related to the procedure. Additionally, some participants doubted the effectiveness of MBS if not followed by lasting behavioral change, believing that the weight would eventually return without personal effort.

Although less frequent, some acknowledged the beneficial (“lifesaving”) impact of MBS, seeing it as a viable option and a tool to improve health and quality of life, and perhaps the most effective or only solution (Table 2: 2.a).

Most participants demonstrated limited or no understanding of the MBS process, with only one showing in‐depth knowledge. Two demonstrated awareness of presurgical requirements such as weight goals, medical appointments, specialists' approvals, and diagnostic testing. One mentioned the importance of social support, noting that lacking emotional or household support could lead to depression and undermine outcomes.

Basic awareness of MBS was reflected in descriptions such as “they cut the stomach” or mentions of surgical bands. Participants knew about general anatomical changes, though lacked detail, with no reference to the hormonal and hypo‐absorption nature of MBS. One participant mentioned the financial burden, including the cost of surgery, supplements, and nutrient‐rich foods—as reasons for preferring other options, such as OMM, which was perceived as more affordable.

Participants knew individuals who experienced both successful weight loss and weight regain after surgery, often attributing recurrence to noncompliance with post‐surgical guidelines. Those with close relatives who had MBS had more detailed knowledge, while others obtained information via secondhand or based on general observations, rather than firsthand experience or formal education. Friends and family were the most common sources of awareness or exposure to MBS.

Participants expressed varying attitudes toward MBS recommendation, ranging from acceptance, conditional openness, and reluctance. Some would consider MBS only if other methods failed or were medically necessary, viewing it as a last resort. A few participants were firmly opposed to MBS, without elaborating on the reasons. Others were receptive but acknowledged emotional or social concerns such as stigma or needing to prepare mentally for public judgment.

Overall, participants' knowledge of MBS, their sources of information, and their attitudes toward MBS recommendations for themselves were closely connected, with limited knowledge and reliance on informal sources contributing to cautious or conditional views of surgery (Table 2: 2.b–d).

Participants offered a range of perspectives on MBS when advising others considering it (Table 2: 2.d), reflecting varying degrees of support, caution, and personal beliefs. A minority expressed supportive views, emphasizing self‐awareness, initiative, and doing what feels right.

A few expressed skepticism or discouragement, citing cost or perceived ineffectiveness, and encouraged exploring non‐surgical options first. For some, MBS was acceptable only after other treatments failed. Other participants offered conditional support, advising others to conduct their own research, seeking medical clearance, and addressing mental health before MBS. These responses reflect the complex and nuanced attitude participants hold toward others' MBS recommendations.

Participants expected positive psychological, social, physical and health‐related changes resulting from weight management (Table 2: 2.e). They anticipated improved mood, confidence, and mental well‐being, greater family engagement and social activity, increased mobility, better chronic condition management, and improved surgical eligibility.

### 3.5 Patient‐Provider Communication

Providers often offered general lifestyle advice without discussing surgical or pharmacologic options (Table 2: 3.a). Some participants had to initiate these conversations, while others reported receiving advice that lacked depth. Two individuals expressed discomfort, citing emotional sensitivity or a lack of trust due to provider turnover. Some noted never discussing obesity with the provider.

Participants identified several factors influencing communication about weight management with healthcare providers (Table 2: 3.b), including trust and continuity of care with a consistent provider. Patient readiness, engagement and initiative to start the conversation were also important. While some preferred direct communication, others felt discomfort discussing obesity. Language barriers and limited integration with nutrition services posed challenges. Although some trusted their providers, conversations often stopped short of exploring treatment options [deleted due to redundancy].

While most participants did not express interest in MBS during their interviews, a small number reflected on considerations, barriers, and informational needs that might encourage them to consider it in the future (Table 2: 3.c–d). These reflections revealed a few emerging considerations related to family planning and post‐pregnancy weight management, having support, and positive peer influence.

Most participants' main concerns about MBS centered around fear of surgical complications and mortality risk. One person touched on a barrier being their personal belief in God's creation and acceptance. In addition to these considerations and barriers, participants expressed a need for more direct, culturally relevant information to support decision‐making about obesity treatment, particularly MBS. They highlighted the importance of understanding eligibility, the surgical process, post‐operative expectations, and required lifestyle changes. Cultural factors—such as food‐centered family gatherings and preparing for behavioral shifts (e.g., smaller portions, avoiding certain foods, refraining from alcohol)—were brought up. Visual or digital formats (e.g., YouTube videos, brochures) and bilingual or interpreted services were preferred to enhance accessibility and trust in care.

## 4 Discussion

This exploratory study identified the perspectives and barriers that contribute to the underutilization of MBS among Hispanic‐Latino adults with obesity living in the U.S., focusing on their perceptions of obesity, obesity management treatments, and their relationship with healthcare providers. Despite the high prevalence of obesity and associated comorbidities in this population as well as the proven effectiveness of MBS in treating obesity and its health‐related complications [8], most participants expressed skepticism, misinformation, and fear about the procedure. While studies on this population and their MBS perceptions are limited, these findings may provide insight about factors that contribute and align with existing literature demonstrating significant racial and ethnic disparities in MBS access and utilization [3, 9, 15].

Participants' perceptions of individuals with obesity and of themselves as well as the causes of obesity were shaped by an interplay of emotional, psychological, behavioral, including physical and dietary, social and health‐related factors. They associated individuals with obesity with negative attributes such as depression, shame, sadness, neglect, tiredness, unhealthy, and others. This illustrates the stigmatization of obesity and highlights the need to focus on minimizing bias and stigma in this community [19, 20]. Obesity was often viewed through a lens of personal responsibility and lifestyle choices though few participants recognized its chronic and relapsing nature. The coexistence of both stigmatizing views and empathetic self‐awareness reflects broader societal narratives that frame obesity as both a personal failure and a public health issue [7]. Weight stigma has been identified as a significant social determinant of health, contributing to adverse health consequences [20]. Therefore, further exploration would be important to verify if the stronger association between weight stigma and decreased mental health [21], disordered eating behaviors [22], and anxiety [23], identified in the general population would also extend to this population as indicated exploratorily here. Interpersonal experiences such as those described here, along with the internalization of stigmatizing beliefs, may contribute to barriers that deter individuals from engaging in health‐promotion behaviors [24], seeking help or considering surgical interventions [15, 25].

The indications of bias, internalized bias, and stigma in this sample challenge the assumption that Hispanic‐Latinos placed less emphasis on body weight concerns and valued larger body sizes than White individuals [26, 27, 28], recognizing that these attitudes may be evolving and that acculturation may play a role in perceptions of obesity and obesity care [17, 29].

MBS was predominantly seen as a last resort with fear of complications and doubts about its long‐term effectiveness. Few participants had a general awareness of the procedure and many lacked accurate or familiarity with the procedure, its requirements, outcomes, or pre‐ and postoperative care—findings echoed in previous research [30]. In our sample, most have learned about MBS from friends and family rather than from their health care providers, often hearing secondhand accounts of adverse outcomes—a factor that may be contributing to conflicting information about MBS [31]. This reliance on informal sources for health information may reflect a gap in culturally sensitive patient education and outreach [14]. Providing MBS information to this community could mitigate these concerns.

Participants reported limited engagement with healthcare providers regarding obesity treatment options, especially MBS and OMM. Conversations were often generic, provider‐driven, and lacked depth. Several participants felt uncomfortable initiating discussions or expressed skepticism or mistrust due to perceived provider insensitivity or language barriers. These dynamics align with prior findings that Hispanic‐Latinos patients often experience less shared decision‐making and lower satisfaction in provider interactions, particularly around weight management [8, 11], perceive disparities in the quality of communication [32], and those who perceived weight‐related judgment from their provider may be less likely to report trust in them [33]. These findings also reflect concerns in the literature regarding the quality and effectiveness of patient‐provider communication about MBS, as well as the role of patient‐centered communication in fostering constructive discussions about MBS [25].

Consistent provider relationships, cultural competency, and bilingual communication emerged as enablers of trust and open dialog—needs that have emerged in previous research [15, 34]. A few participants expressed a need for more personalized, culturally relevant guidance and informational materials—preferably in multimedia formats and in Spanish. Incorporating visual tools and community‐based education could help demystify MBS and counteract misinformation, increasing acceptability among hesitant patients [14, 31].

These findings underscore the importance of enhancing provider training on culturally sensitive communication, developing multilingual, accessible, and culturally relevant educational materials, strengthening community engagement to build trust and increase awareness of MBS, and addressing systemic inequities in insurance and referral processes.

The strengths of this study include its valuable insights into weight bias, stigma, and internalized bias, as well as the influence of culture on obesity care. This research provides an in‐depth exploration of the perspectives of Hispanic–Latino adults living with obesity, highlighting the sociocultural factors that shape their decision‐making regarding treatment. By amplifying voices directly from the community, this study contributes meaningful qualitative data to an area that remains underexplored in obesity research. The findings identified key opportunities to address fears, misconceptions, and skepticism surrounding obesity and the safety of MBS. Moreover, the results offer important direction for the development of culturally relevant interventions aimed at improving access, enhancing patient–provider communication, and ultimately reducing disparities in obesity care.

The present study has several limitations. Although participants reported obesity‐related conditions and unsuccessful attempts to manage their obesity, the sample primarily consisted of individuals with Class I obesity. In addition, the sample was predominantly composed of middle‐aged women. While this demographic reflects the general MBS population [35], increasing the number of men would provide a broader range of perspectives and improve representativeness. Furthermore, the exact number of individuals initially approached for recruitment is unknown, as the community center director distributed flyers and maintained the list of eligible participants. Only the 14 individuals who met the eligibility criteria and completed the interviews were documented. It is possible that additional community members expressed interest but were not eligible or not tracked, which may have introduced some selection bias and limited diversity.

## 5 Conclusion

Despite qualifying for MBS, Hispanic‐Latino adults with obesity face a convergence of barriers—psychosocial, cultural, informational, and systemic—that influence their treatment decisions. Participants' narratives revealed that low utilization of MBS in this group is not simply a matter of access but of trust, knowledge, and cultural relevance. Culturally tailored interventions, increased provider awareness and rapport with this community are critical for closing this gap. By centering the voices and lived experiences of Hispanic‐Latino individuals, future efforts can move toward greater utilization and access of obesity care.

## Author Contributions

All listed authors contributed substantially to this manuscript and approved the final submitted version. Specifically, Viviane was the primary contributor and was involved in all aspects of the study, including conception, methodology, data collection, data analysis, interpretation, and manuscript writing. Ceren supervised all stages of the study and contributed significantly to ensuring methodological rigor throughout as well as to writing and critical revision of the manuscript. Kathleen provided subject‐matter expertise, contributed to the interpretation of results, and offered critical feedback and revision throughout the research and writing process.

## Funding

The authors have nothing to report.

## Conflicts of Interest

The authors declare no conflicts of interest.
